# Mating success of resident versus non-resident males in a territorial butterfly

**DOI:** 10.1098/rspb.2007.0311

**Published:** 2007-05-01

**Authors:** Martin Bergman, Karl Gotthard, David Berger, Martin Olofsson, Darrell J Kemp, Christer Wiklund

**Affiliations:** 1 Department of Zoology, Stockholm University106 91 Stockholm, Sweden; 2 School of Tropical Biology, James Cook UniversityPO Box 6811, Cairns, Queensland 4870, Australia

**Keywords:** Lepidoptera, contest success, mate locating behaviour, female choice, mate choice, butterfly vision

## Abstract

Male–male competition over territorial ownership suggests that winning is associated with considerable benefits. In the speckled wood butterfly, *Pararge aegeria*, males fight over sunspot territories on the forest floor; winners gain sole residency of a sunspot, whereas losers patrol the forest in search of females. It is currently not known whether residents experience greater mating success than non-residents, or whether mating success is contingent on environmental conditions. Here we performed an experiment in which virgin females of *P. aegeria* were allowed to choose between a resident and a non-resident male in a large enclosure containing one territorial sunspot. Resident males achieved approximately twice as many matings as non-residents, primarily because matings were most often preceded by a female being discovered when flying through a sunspot. There was no evidence that territorial residents were more attractive *per se*, with females seen to reject them as often as non-residents. Furthermore, in the cases where females were discovered outside of the sunspot, they were just as likely to mate with non-residents as residents. We hypothesize that the proximate advantage of territory ownership is that light conditions in a large sunspot greatly increase the male's ability to detect and intercept passing receptive females.

## 1. Introduction

As with many flying insects, male mate location in butterflies can be divided into two broad and generally species-specific categories: ‘perching’ and ‘patrolling’ (Scott 1974; Wiklund 2003). In patrolling species males spend the major part of their life actively searching for females, but in perching species the roles are reversed and females assume the role of actively searching for a mate. In the latter case, males are typically faithful to their perching sites and attempt to exclude other males from their site (i.e. they are ‘territorial’). Territories are defended by means of largely non-contact aerial interactions in which the two combatants circle or hover near each other for a period of time before one male ‘gives up’ and is chased from the site (Davies 1978; Wickman & Wiklund 1983; Kemp & Wiklund 2001, 2004). In a seminal paper on territory defence in the speckled wood butterfly, *Pararge aegeria*, Davies (1978) observed that it was virtually always the resident male that prevailed in these contests over site ownership. In the following years, considerable effort has been expended on understanding whether certain physical and/or physiological characteristics are also associated with contest success (and hence residency), including body size (Kemp 2000; Takeuchi 2006), body temperature (Stutt & Willmer 1998; Kemp & Wiklund 2004), energy reserves (Kemp 2002*a*) and flight morphology (Kemp *et al.* 2006). As yet, however, there is no strong evidence for a causal role of any of these factors, leaving Davies' (1978) observation that ‘the resident always wins’ as the best proximate predictor of contest outcome in butterflies (Rosenberg & Enquist 1991; Kemp 2000; Kemp & Wiklund 2001, 2004; although see the work of Kemp (2000, 2002*b*, 2003) regarding the effects of age).

Since butterfly territories typically do not contain an abundance of larval or adult resources, the consensus is that they serve as rendezvous places where the sexes meet. However, in spite of numerous studies, there are few actual observations of matings occurring in contested territories (although see Wickman (1985*b*)). The putative function of territories as sexual rendezvous sites is therefore based largely upon circumstantial evidence (cf. Davies 1978; Wickman & Wiklund 1983; Shreeve 1984; VanDyck *et al.* 1997; Jones *et al.* 1998; Kemp 2000, 2001). This is disturbing, especially in view of the common observation that suitable territories are often in short supply, which means that competition can be intense and that part of the male population forms a subpopulation of non-territorial floaters. In order to accurately identify and understand the selective pressures on males in these populations, it would be useful to know exactly how territorial ownership translates into increased mating success.

In a system in which the females actively search for males, male mating success is conceivably influenced by at least two factors: (i) the degree to which male perching sites (i.e. territories) coincide with female dispersion and (ii) any female preferences for particular male character traits. In the first case, male mating success is likely to be influenced by how well territory location coincides with female movement patterns (Wickman & Rutowski 1999). Receptive females are rarely expected to be randomly distributed in space and time (Parker 1974), and sites defended by territorial males should therefore be related to a high probability of encountering receptive females. Males of territorial butterfly species may defend areas associated with egg-laying females (Baker 1972; Courtney & Parker 1985; Rosenberg & Enquist 1991; Lederhouse *et al.* 1992), female food resources (Suzuki 1976; Fischer & Fiedler 2001) or female emergence sites (Deinert 2003). Males may also defend well-defined topographical or physical structures that are devoid of obvious resources, but function as easily identifiable landmark structures, such as gullies (Cordero & Soberon 1990), sunspots on the forest floor (Davies 1978; Wickman & Wiklund 1983), elevations and hilltops (Shields 1967; Lederhouse 1982; Alcock 1987), or trees and bushes (Wickman 1985*a*). Sometimes several males may defend territories close to each other effectively forming a lek at such landmark sites (Wickman 1985*a*). In a number of hilltopping and lekking species, it has been convincingly shown that virgin females actively visit sites where males are located to mate (Shields 1967; Wickman 1985*b*, 1988; Wickman & Jansson 1997).

In the second case, aside from the process of physically locating a female, a male's likelihood of actually mating may depend upon whether females prefer particular character traits. In birds, it has been convincingly demonstrated that females can have a preference for particular male ornaments (cf. Andersson 1994), and in butterflies there is now abundant evidence that female mate choice is influenced by both visual and olfactory stimuli (Stride 1958; Silberglied & Taylor 1978; Fordyce *et al.* 2002; Ellers & Boggs 2003; Sweeney *et al.* 2003; Andersson *et al*. 2007; Costanzo & Monteiro 2007; Kemp 2007). Moreover, in some territorial species, such as the nymphalines *Polygonia c-album*, *Aglais urticae*, *Inachis io* and *Vanessa atalanta*, the courtship phase is extraordinarily extended (typically a male follows a female for several hours before mating is initiated), and it would seem that only males with the ability for high performance flight can ever successfully mate (Baker 1972, 1983, 1984; Bitzer & Shaw 1979). Thus, it is possible that males could experience selection for the ability to select and defend appropriate territories as well as for the possession of physical or physiological traits preferred by females.

In this study, we used *P. aegeria* as an experimental model in order to address three related issues regarding territoriality, mate selection and mating success in butterflies. First, we set out to experimentally test the fundamental assertion that territorial residents achieve higher mating success than non-residential floaters. As noted above, this assertion underpins our present understanding of the evolution of butterfly territoriality, yet observational data on this point are extremely scarce. Second, we attempted to assess whether receptive virgin females of *P. aegeria* possess an active preference for flying into large sunspots. If so, and if females mate more or less indiscriminately, this would explain an obvious advantage of territorial residents over non-residents. Last, we set out to assess whether virgin receptive females may have preferences for particular male character traits associated with territorial residency. We answered these questions by releasing virgin females into a large experimental enclosure containing two males who had previously had the opportunity to compete for a single sunspot territory in the enclosure (and had thus settled into well-defined resident and non-resident ‘floater’ roles).

## 2. Material and methods

### (a) Experimental cages

We conducted the experiments in a large outdoor cage, located at Kronängen (approx. 100 km south of Stockholm in central Sweden). We used one cage divided into two identical parts that were semi-cylindrically shaped with a 15×8 m base and 4 m radius, covered with a 32% UV-absorbing shade-cloth cover overlaid with a green plastic tarpaulin. We removed a 2×2 m section of the tarpaulin to create a large sunspot that tracked across the cage floor from 09.00 to 17.00, and cut a series of smaller (0.2×0.2 m) holes to create a mosaic of smaller sun flecks. The male butterflies quickly and consistently recognized the large sunspot as a suitable territorial site. The floor of the cage consisted of native, unmown grass, and we also placed out artificial plastic Christmas trees near the large sunspot to further simulate a real forest habitat.

### (b) The trials

The experiments were performed in June and August 2004 and in June and July 2006. We used a population of *P. aegeria* originating from Madeira, Portugal, reared at the laboratory at the Department of Zoology, Stockholm University, and brought to Kronängen in ice-filled coolers. The experiments were conducted in two steps. First, two males were taken out of the cooler and simultaneously introduced into the cage, each placed on a separate moistened cotton wool bud (15% sugar solution), and were never handled directly. The butterflies perched on the cotton wool bud for several minutes, to feed and warm up, prior to flying around the enclosure. Upon meeting, the two newly introduced males typically engaged in several aggressive interactions and a dominance asymmetry between the two males became established. We observed a minimum of three contests before we considered the dominance relationship to be established. Dominant males always settled in the large sunspot, and controlled this area plus a variable extent of the immediate surrounds. These males also performed scouting flights around in the cage, with subsequent male–male encounters always ending with the dominant male chasing the subordinate (i.e. the dominance relationship, once established, was always preserved).

Once the residency asymmetry was established, we released a virgin female (using methods as per the males) into a small sunspot equidistant between the two males. Females used in the experiments had eclosed between 1 and 26 days earlier (mean=10.5; s.d.=5.1), and were fed every 4–5 days and held in a cold (10°C) room to slow down their physiological ageing as much as possible prior to the experiment. We recorded whether the female mated with the resident or the non-resident male and the time elapsed between female introduction and mating. We additionally recorded the course of events, such as courtship, rejection of males by the female, and whether the female was detected by the mating male in, or outside of, the large sunspot. In the 57 trials done in 2006, out of the 127 trials studied, we recorded every single landing the female did until she was mated. The landings were classified into three categories: landings in a large sunspot, in a small sunspot or in the shade. Cage temperature and time of the day was recorded for each trial. Data on all variables were not available for all individuals and sample sizes might therefore vary in the different statistical tests. In the statistical analysis of the effect of environmental variables on the association between male residency and mating success, we used data from all mated males in a model with contest outcome (resident/non-resident) as a dependent variable and temperature, time of the day and female age as continuous factors.

## 3. Results

### (a) The territorial contest

We conducted 127 mating trials. The outcome of the territorial contests between the two males in each trial was affected neither by male body mass (paired *t*-test: *t*_84_=1.009, *p*=0.316) nor age (paired *t*-test: *t*_87_=1.453, *p*=0.150), and once the dominance relationship was settled, we did not observe a single case of reversal.

### (b) Mating success

All of the 127 virgin females that were used in the experimental trials mated, and the time until mating varied from 1 to 74 min. Residents (i.e. males that had won the territorial contest(s)) were more successful in obtaining matings than losers: residents mated with the female in 82 out of the 127 trials (figure 1; two-sided binomial test: *Z*=3.28, *p*=0.001). Time elapsed from female introduction until mating was influenced by female age, with older females mating more quickly (linear regression: *r*^2^=0.15; ANOVA: *F*_1,53_=9.16, *p*=0.004), but this variable did not affect the association between male residency and mating success (GLZ: Wald stat.=1.152, d.f.=1, *p*=0.283). The mating advantage of residency was also unrelated to temperature during the trials (which ranged from 18 to 30°C; GLZ Wald stat.=0.363, d.f.=1, *p*=0.547) and time of the day (which ranged from 10.05 to 16.35; GLZ Wald stat.=1.439, d.f.=1, *p*=0.230).

### (c) Female rejection of courting males

There was no significant difference between residents and non-residents in the probability of being rejected by a female (*n*_rejected resident_=12; *n*_accepted resident_=43; *n*_rejected non-resident_=12; *n*_accepted non-resident_=21; Fisher's exact two-tailed test: p=0.148). Females mated with the first male that courted her in 64 trials and rejected the first courting male in 24 cases. When the female rejected the first courting male, the male that she eventually mated with was equally often a non-resident as a resident male (*n*_resident_=12; *n*_non-resident_=12).

### (d) Female detection

Virtually, all male–female interactions were triggered by a perching male discovering a female in flight close by. Matings were more often preceded by a female being discovered when flying through the large sunspot than outside of it (table 1; *n*_large sunspot_=54; *n*_outside large sunspot_=31; two-sided binomial test: *Z*=2.495, *p*=0.013). When the female was detected in the large sunspot, the mating male was more often the resident male (*n*_resident_=48; *n*_non-resident_=6; two-sided binomial test: *Z*=5.716, *p*<0.001), while when the female was detected outside the large sunspot, non-resident males were as likely to mate with the female as resident males (*n*_resident_=14; *n*_non-resident_=17; two-sided binomial test: *Z*=0.539, *p*=0.590).

### (e) Female landings

The females landed more often in the smaller sun flecks than in the large sunspot (figure 2 and table 1; Wilcoxon matched-pair test, Bonferroni corrected: *n*=57, *T*=104, *Z*=4.345, *p*<0.001) or the shade (table 1; Wilcoxon matched-pair test, Bonferroni corrected: *n*=57, *T*=183, *Z*=3.641, *p*<0.001). There was no difference in the number of landings between the large sunspot and the shade (table 1; Wilcoxon matched-pair test, Bonferroni corrected: *n*=57, *T*=273.5, *Z*=1.835, *p*=0.067). To test whether landings of females were more frequent in any of the three categories than expected by chance, the same analyses were done on landings per square metre. The landing frequency of females was significantly higher in the small sun flecks than in the large sunspot (table 1; Wilcoxon matched-pair test, Bonferroni corrected: *n*=57, *T*=127, *Z*=4.728, *p*<0.001) or in the shade (table 1; Wilcoxon matched-pair test, Bonferroni corrected: *n*=57, *T*=98, *Z*=5.026, *p*<0.001). There was no difference in landing frequency between the large sunspot and the shade (table 1; Wilcoxon matched-pair test, Bonferroni corrected: *n*=57, *T*=469, *Z*=0.303, *p*=0.761).

## 4. Discussion

Here we show that resident males achieve higher mating success than non-residents in a territorial mating system, with resident males being almost twice as successful as non-residents. This pattern was environmentally stable and was not affected by the temperature or time of the day. There was little evidence that females actively preferred to mate with resident males *per se*; in approximately 37% of the trials, females rejected the first courting male and they rejected residents and non-residents equally often. Moreover, there was no association between residency and mating success after a first rejection by a female. The majority of these matings were preceded by a female being discovered when flying into the large sunspot. On these occasions, the mating male was most often the resident, but when the female was detected outside the large sunspot, non-residents were as likely to mate with the female as were residents. The virgin females did not appear to have a preference for visiting the large sunspot and landed most often in a small sun fleck, and landing frequency per square meter was also significantly higher in the small sun flecks than expected by chance. It should be noted that landings in the large sunspot were more likely to lead to a mating and the termination of the trial, and we may therefore have underestimated landings in the large sunspot. Nevertheless, our data give no support for a strong female preference for flying to the large sunspot. Hence, our interpretation of how the *P. aegeria* mating system works is that resident males do not have any traits that make them more attractive to females than non-residents, but that females are most easily discovered, and possible to pursue, when flying through a large sunspot, which means that a resident male has priority and is more likely to discover, court and mate with a female than a non-resident male.

### (a) Residency and male mating success

What maintains territoriality in butterflies? The most obvious explanation would be that residents have higher mating success than non-residents, which has been hypothesized by several authors (Davies 1978; Lederhouse 1982; Wickman & Wiklund 1983). Others have hypothesized that relative mating advantage of residents versus non-residents is contingent on environmental factors such as temperature, microclimate and habitat structure (Shreeve 1984; VanDyck *et al.* 1997). Regardless, matings in the wild are notoriously difficult to observe, and consequently a clear evaluation of these hypotheses has been severely hampered by lack of empirical support. Part of the reason why natural matings are so difficult to observe is that the majority of butterfly females mate soon after eclosion, and typically the overwhelming majority of active females in a population have already mated (Wiklund & Fagerstro¨m 1977). For example, only 13 out of 997 (i.e. 1.3%) wild-collected pierid and satyrine female butterflies were unmated (Wiklund & Forsberg 1991). As a corollary, the majority of observed male courtships of females in nature end without mating, and so out of 117 observed courtships of female *Leptidea sinapis* not a single one resulted in mating (Wiklund 1977). For these reasons, the only way to get a good measure of male mating success is to follow receptive virgin females, which usually necessitates release of laboratory-reared individuals. Using this method, we have shown that resident males of *P. aegeria* enjoy higher mating success than non-residents, and that residents achieved, on average, twice as many matings under the conditions that prevailed in our experiment. However, although residency furnished a greater reproductive pay-off, non-residents were equally successful in mating with females that were not intercepted by a male when flying through a sunspot. Our results therefore indicate that while there is a residency-linked asymmetry in likely male mating success, territorial ownership is not absolutely necessary for a male to achieve a mating. This may help explain the variance in male willingness to engage in time-consuming territorial contests and the fact that all males are not apparently motivated to fight ‘to the death’ over ownership (i.e. the absence of Grafen's (1987) ‘desperado effect’). In our experiments, the proportion of residents to patrollers was equal, but under field conditions the proportion of patrollers is likely to change as population density increases in the later part of the season (Heath *et al.* 1984). Although residents had a higher mating success in our experiments, the proportion of females that are mated by patrolling males in the field is likely to increase, as the probability of a female being intercepted by a patroller increases. Hence, it is possible that the relative benefit of being a resident or a patrolling male is in part influenced by population density, but whether the *per capita* benefit of residents and patrollers is influenced by density is a complicated issue that warrants further investigation.

### (b) Female mate choice

Why do resident males achieve higher mating success than non-residents? This pattern could result from resident males being somehow more attractive than non-residents, or if they possess some powers or attributes that non-residents simply do not have. It is well known that female choice in butterflies can be guided by visual as well as olfactory cues (Stride 1958; Silberglied & Taylor 1978; Fordyce *et al.* 2002; Ellers & Boggs 2003; Sweeney *et al.* 2003; Andersson *et al*. 2007; Costanzo & Monteiro 2007; Kemp 2007). However, our observations that non-residents were as likely to mate with females discovered outside of the large sunspot and that residents were as likely to be rejected as were non-residents suggest that female mate choice *per se* does not underlie the higher mating success of resident males. What about a conceivable difference in flight and manoeuvring performance between residents and non-residents? Although all of the females used in the experiments were virgin, their behaviour when discovered by a male differed. Typically, females that accepted the first courting male (64 out of 88) made a short flight and landed on the Christmas tree and mated after a brief courtship. Females that did not accept the first male (24 out of 88), however, actively appeared to attempt to escape by outflying the engaging male. Males pursued females as closely as possible during these female ‘escape’ flights, but many eventually lost contact. It should also be noted that a limited number of females that did mate with the first courting male also launched a rapid escape flight, and these females seemed willing to mate only after having ‘tested’ the male's manoeuvring ability. If so, and if male residency is correlated with male flight manoeuvrability, this could explain part of the resident male mating advantage. Such an apparent test of male manoeuvring ability is a customary part of the mating system of nymphaline butterflies such as *P. c-album*, *A. urticae*, *I. io* and *V. atalanta* (Baker 1972, 1983, 1984; Bitzer & Shaw 1979; C. Wiklund personal observation); however, the experimental data from our experiments do not provide any evidence for such a coupling of male manoeuvring ability, contest success and mating success. Further experimentation will be ultimately required to resolve this issue.

### (c) Female dispersal and male mating success

Why were most of the matings in our experiment preceded by a female being discovered flying through a large sunspot? This pattern could either be explained by a female preference for large sunspots or by females being more likely to be discovered, successfully pursued and courted after flying through such a sunspot. Wickman & Rutowski (1999) argued that selection will favour female behaviour that minimizes time spent without sperm and time and energy cost of mate location. Indeed, Wickman *et al*.'s (1995) study of mate searching of virgin female *Coenonympha pamphilus* showed that receptive females actively flew towards landmarks where male territories could be predictably located. Moreover, it has been shown in several butterfly species that virgin females actively solicit courtship and approach males, or use visual displays to attract mates (Rutowski 1980; Wiklund 1982; Wickman 1986). Hence, in a territorial system such as that of *P. aegeria,* in which males are most predictably found in large sunspots and virtually all females mate only once in their lifetime (mean number of spermatophores/female in the field=1.04; Wickman & Wiklund 1983), a female preference for such places could be expected. However, our results suggest that females of *P. aegeria* do not seem to have such a preference. Therefore, an alternative explanation for why most of the matings in our experiment were preceded by a female flying through a large sunspot could be that male discovery of females is facilitated when females do fly through a large sunspot.

Mate location in butterflies is almost exclusively based upon vision. Perching males have to detect a passing object that is often moving quite rapidly and is often juxtaposed against a complex background. The ability to efficiently detect a moving object depends on acuity of the eye, the rate and motion of the flying object, the background and ambient levels of illumination (Rutowski 2003). Several of these factors are likely to vary with mate searching strategy in *P. aegeria*, with perching males facing a more illuminated environment and potentially more contrasting visual backgrounds than patrolling males. Studies on other territorial butterfly species, addressing the importance of vision for mate location while perching, have indeed found adaptations in eye morphology (Rutowski 2000*b*), perching locations and posture (Ravenscroft 1994; Rutowski 2000*a*), which are believed to increase their chances of visually detecting potential mates. Our results are consistent with the idea that the main driving factors behind greater resident male success rates relate to territory ownership rather than the attractiveness of the incumbent. Given this, it is conceivable that the value of using large sunspots as territories in forest landscapes is that the light conditions are more conducive to visually detecting flying females. Furthermore, the size of the sunspot may be highly important such that a minimal area of bright illumination is required for a male to accurately calculate the female flight path and his subsequent interception trajectory. This hypothesis is eminently testable and offers an attractive focus for further study for the advancement of our understanding of butterfly territoriality in forest habitats.

## Figures and Tables

**Figure 1:**
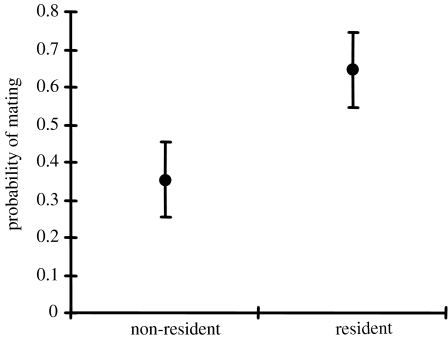
Mating success of resident and non-resident males, respectively, in 127 mating trials during which a female exercised mate choice between a resident male that controlled a 2×2 m sunspot territory and a non-resident male without sunspot territory; values are given with a 95% CI.

**Figure 2:**
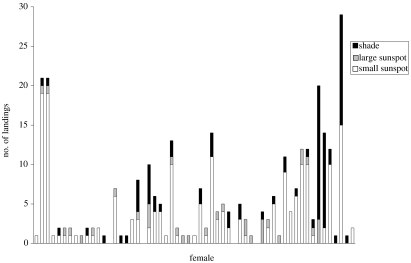
Female landing frequencies in three categories, the large sunspot, the small sunspot or in the shade, for 57 females during June and July 2006. In the cases where no landing was recorded, the female was discovered and mated immediately after first takeoff.

**Table 1:** The average number of landings (±s.d.) for a female, the average landing frequency per area (±s.d.) and the proportion of mated females discovered in three habitat categories, during 57 trials in 2006.

	small sunspot	large sunspot	shade
landings	3.32 (±4.59)	0.58 (±0.80)	1.47 (±3.27)
landings per square metre	0.99 (±1.38)	0.14 (±0.20)	0.20 (±0.45)
proportion of mated females discovered (%)	26	63	11
